# Canine Hepacivirus NS3 Serine Protease Can Cleave the Human Adaptor Proteins MAVS and TRIF

**DOI:** 10.1371/journal.pone.0042481

**Published:** 2012-08-01

**Authors:** Mariona Parera, Gloria Martrus, Sandra Franco, Bonaventura Clotet, Miguel Angel Martinez

**Affiliations:** Fundació irsiCaixa, Hospital Universitari Germans Trias i Pujol, Universitat Autònoma de Barcelona (UAB), Badalona, Spain; University of Tennessee Health Science Center, United States of America

## Abstract

Canine hepacivirus (CHV) was recently identified in domestic dogs and horses. The finding that CHV is genetically the virus most closely related to hepatitis C virus (HCV) has raised the question of whether HCV might have evolved as the result of close contact between dogs and/or horses and humans. The aim of this study was to investigate whether the NS3/4A serine protease of CHV specifically cleaves human mitochondrial antiviral signaling protein (MAVS) and Toll-IL-1 receptor domain-containing adaptor inducing interferon-beta (TRIF). The proteolytic activity of CHV NS3/4A was evaluated using a bacteriophage lambda genetic screen. Human MAVS- and TRIF-specific cleavage sites were engineered into the lambda cI repressor. Upon infection of *Escherichia coli* cells coexpressing these repressors and a CHV NS3/4A construct, lambda phage replicated up to 2000-fold more efficiently than in cells expressing a CHV protease variant carrying the inactivating substitution S139A. Comparable results were obtained when several HCV NS3/4A constructs of genotype 1b were assayed. This indicates that CHV can disrupt the human innate antiviral defense signaling pathway and suggests a possible evolutionary relationship between CHV and HCV.

## Introduction

The origin of hepatitis C virus (HCV) infections in humans has remained unknown, because related animal virus homologs had not been identified [1], [2]. Recently, a *Flaviviridae* RNA genome that was isolated from domestic dogs with respiratory illness was found to be the virus most closely related to HCV [3]. This *Flaviviridae* agent is known as canine hepacivirus (CHV). The discovery of CHV may shed light on the origin of HCV, and may also serve as a new model system with which to study this deadly human virus. CHV-like viruses (also known as nonprimate hepaciviruses [NPHV]) were also recently identified in horses [4]. HCV belongs to the genus hepacivirus, one of the four genera in the family *Flaviviridae* [2]. HCV infects more than 170 million people worldwide (http://www.who.int/mediacentre/factsheets/fs164/en/) and is one of the leading causes of liver cirrhosis and failure [5]. Although the discovery of the close homology between CHV and HCV is intriguing, there are barriers preventing viral transmission across species. Cross-species transmission of CHV to humans would most likely require evasion of the human cellular innate immune response, which leads to type I interferon production through RNA composition–dependent activation of retinoic acid inducible gene-I (RIG-I) and toll-like receptor (TLR) [6].

A number of host-encoded restriction factors can protect human cells from viral infections. A well characterized model is retroviruses. The human genome has evolved a constellation of antiviral genes with the potential to target retroviruses. These genes are part of the innate immune system, and in the retrovirus literature, they are often called restriction factors. Well-characterized restriction factors are APOBEC3G [7], TRIM5-α proteins [8], tetherin (BST2) [9], and SAMHD1 [10], [11], which inhibit human immunodeficiency virus (HIV) propagation at various steps in its replication cycle. The evolution of the genes encoding these restriction factors affected the adaptation of HIV to humans in the current pandemic, and the adaptation of other primate lentiviruses to their modern hosts. In the case of HCV, signalling pathways leading to type I interferon production are the first line of defence employed by the host to combat viruses and represent a barrier that invading viruses must overcome in order to establish infection [12], [13]. A major strategy employed by HCV to subvert the host innate immune response is disruption of the RIG-I and TLR-signalling pathway through HCV NS3/4A protease cleavage of the adaptor proteins MAVS and TRIF [14], [15]. MAVS from multiple primates are resistant to inhibition by the HCV NS3/4A protease [16]. This resistance maps to single changes within the protease cleavage site in MAVS, which protect MAVS from getting cleaved by the HCV NS3/4A protease. These changes were likely driven by ancient hepaciviruses, providing insights into hepaciviral infections over the course of primate evolution [16].

Because the propagation of CHV and NPHV in humans would most likely have had to overcome the human innate host defence mediated by type I interferon, we hypothesized that CHV NS3/4A protease could specifically process the human adaptor proteins MAVS and TRIF. To test this hypothesis, we coexpressed the CHV NS3/4A protease with specific MAVS and TRIF cleavage sites that are known to be processed in vivo by the HCV NS3/4A protease. Using this approach, we demonstrated that the CHV NS3/4A protease can cleave human MAVS and TRIF.

## Results

**Figure 1:**
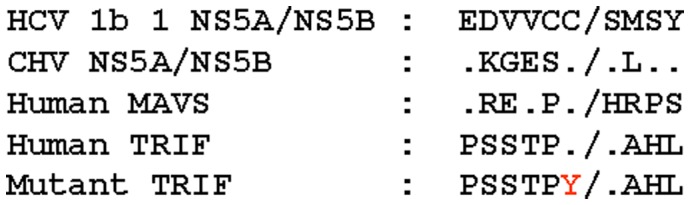
Alignment of peptide junctions present in MAVS, TRIF, and viral polyproteins of HCV and CHV, and cleaved in *trans* by their respective protease. A mutated residue generated by site-directed mutagenesis is marked in red.

**Figure 2:**
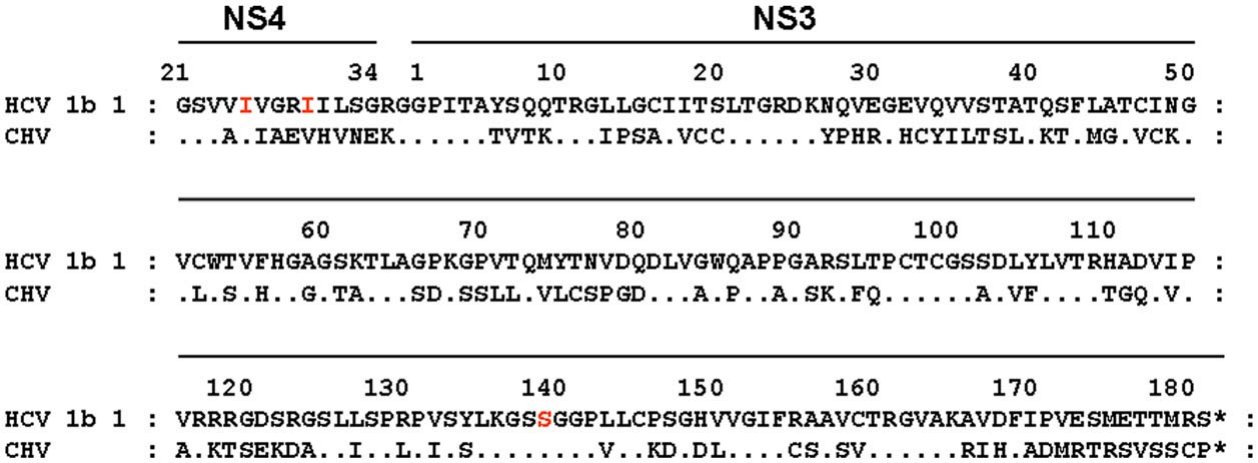
Amino acid sequences of the NS3/4A protease constructs engineered in this study. HCV (sample 1) and CHV NS3/4A protease constructs contain NS4 residues 21–34 fused in frame via a short linker to the amino terminus of the NS3 protease domain (residues 1–181). It was previously demonstrated that the kinetic parameters of a single-chain protein containing the NS4A cofactor and the HCV NS3 protease are identical to those of the bona fide NS3/4A (NS3 residues 1 to 631 and NS4A residues 1 to 54 protein complex generated in eukaryotic cells (5, 38). Asterisks represent the stop codons. Mutated residues generated by site-directed mutagenesis are marked in red.

The catalytic activity of CHV NS3/4A protease was assayed using a bacteriophage lambda–based genetic screen. We previously demonstrated that the bacteriophage lambda–based genetic screen employed here can be used to characterize site-specific proteases, including HCV NS3/4A, HIV, and respiratory syndrome (SARS) coronavirus proteases [17], [18], [19], [20], [21], [22], [23], [24]. This genetic screen is based on the bacteriophage lambda cI-cro regulatory circuit, in which the viral repressor cI is specifically cleaved to initiate the switch from lysogeny to lytic infection [25]. This genetic screen is a simple alternative approach to the purification and characterization of expressed proteases by in vitro classical methodologies. The specific cleavage sites engineered in the lambda cI repressor and assayed in this study are depicted in Fig. 1. These sites included specific MAVS and TRIF cleavage sites known to be processed in vivo by the HCV NS3/4A protease, as well as CHV and HCV NS5A/NS5B viral polyprotein cleavage sites. A mutated version of the TRIF cleavage site was also included as a control. The target specificity for the MAVS and TRIF cleavage sites was tested by coexpressing them with CHV or HCV NS3/4A protease constructs. The NS3/4A protease constructs contained NS4 residues 21–34 fused in frame via a short linker to the amino terminus of the NS3 protease domain (residues 1–181) (Fig. 2). The CHV NS3/4A protease construct was chemically synthesized following the nucleotide sequence reported by Kapoor et al. [3].

**Figure 3:**
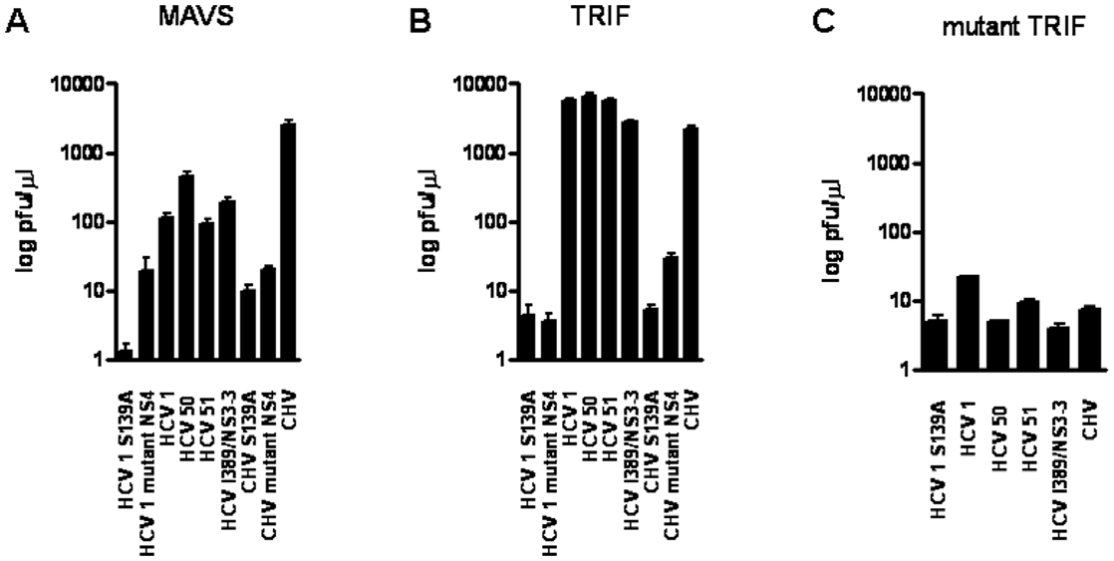
Comparative catalytic efficiencies of HCV and CHV NS3/4A proteases based on (A) MAVS, (B) TRIF, and (C) mutant TRIF cleavage. The graph illustrates the resulting lambda phage titer (in PFU per microliter) after selective growth of lambda phage in *E. coli* cells coexpressing the NS3/4A protease constructs and the lambda cI repressor expressing either MAVS, TRIF, or mutant TRIF cleavage sites. As shown, selection in cells coexpressing the NS3/4A protease construct containing the inactivating S139A mutation or the mutated version of NS4 severely compromised the replication of lambda phage. Similarly, the phage did not replicate when a mutated version of TRIF was coexpressed. Values are the means ± standard deviations (error bars) of at least three experiments.

Upon infection of *E. coli* cells coexpressing the lambda cI repressor with either MAVS or TRIF cleavage site and a CHV NS3/4A construct, lambda phage replicated up to 2,000-fold more efficiently than in cells expressing a CHV protease variant that included a substitution in catalytic residue S139 (Fig. 3A and 3B). Equivalent results were obtained when four HCV NS3/4A of genotype 1b were assayed. Three assayed HCV NS3/4A proteases were isolated from three treatment-naïve HCV-infected patients [18], [24]. The fourth HCV NS3/4A protease was amplified from the HCV subgenomic replicon I389/NS3-3 [26].

To investigate the influence of the NS4 protein on the catalytic efficiency of CHV NS3 protease, the CHV NS4 residues I25 and V29 were replaced with A residues (Fig. 1). HCV NS4 residues 25 and 29 are critical for NS4 activation of HCV NS3 protease [18], [27]. Similar to HCV NS3 protease, a mutated version of CHV NS4 reduced the catalytic efficiency of the CHV NS3 protease, indicating that the CHV NS4 peptide is essential for efficient processing of MAVS and TRIF cleavage sites (Fig. 3A and 3B). Nevertheless, the effect of the mutated NS4 residues 25 and 29 on CHV NS3/4A protease activity was less drastic than that observed on HCV NS3/4A protease (Fig. 3B) (see below).

We further tested the target specificity of the MAVS and TRIF cleavage sites by analyzing a control mutant TRIF target site. A mutant TRIF cleavage site was constructed in which the C residue at the P1 position was substituted with a Y residue (Fig. 1). As shown in Fig. 3C, the mutant TRIF cleavage site prevented phage replication, indicating that TRIF processing was specifically mediated by CHV NS3/4A protease.

**Figure 4:**
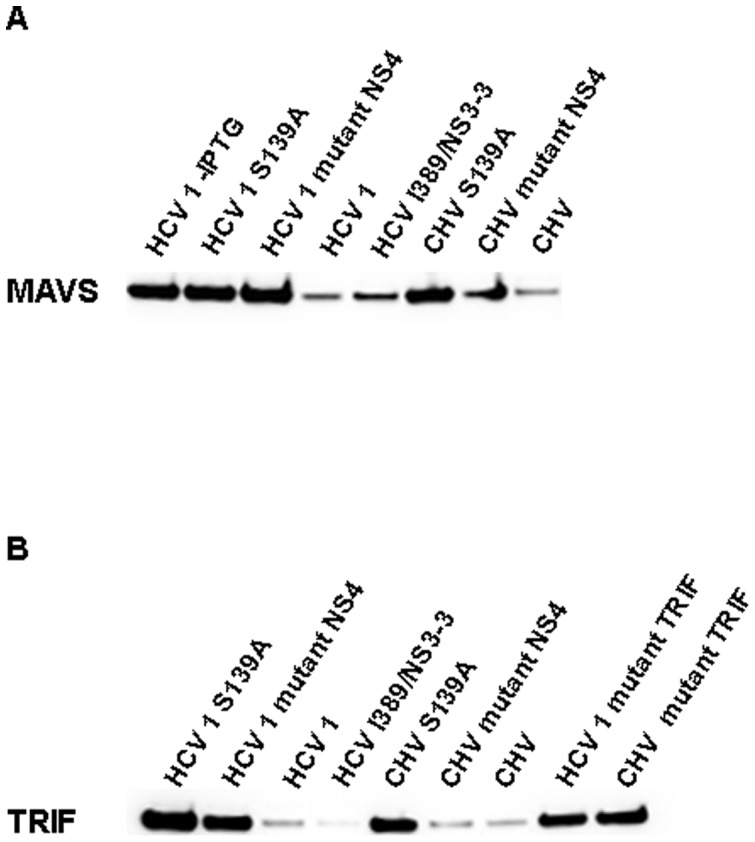
Expression of the CHV or HCV NS3/4A protease resulted in nearly complete cleavage of the lambda cI repressor with either MAVS (A) or TRIF (B) cleavage site. Expression of the protease was induced with IPTG for 3 h. The Western blot proved that the lambda cI repressor with either MAVS or TRIF cleavage site was not cleaved by NS3/4A proteases that included a substitution in catalytic residue S139. Similarly, a control mutant TRIF target site (Fig. 1) was not cleaved by wild-type NS3/4A proteases. The HCV 1 NS3/4A protease was also tested without IPTG.

Western blot analysis also demonstrated (Fig. 4) that the expression of the CHV NS3/4A protease resulted in nearly complete cleavage of the lambda cI repressor with either MAVS (Fig. 4A) or TRIF cleavage site (Fig. 4B). Expression of NS3/4A proteases that included a substitution in catalytic residue S139 (Fig. 4) completely abolished the cleavage of lambda cI repressor with either MAVS or TRIF cleavage site. Expression of NS3/4A proteases with a mutated version of NS4 partially abolished wild-type MAVS or TRIF repressor cleavage (Fig. 4); specifically, mutations in NS4 had less impact in the activity of CHV NS3/4A protease than in the activity of HCV NS3/4A protease (Fig. 4B). As expected, a control mutant TRIF target site (Fig. 1) was not cleaved by wild-type NS3/4A proteases. Overall, these results demonstrate that CHV NS3/4A protease is able to specifically cleave the human adaptor proteins MAVS and TRIF.

**Figure 5:**
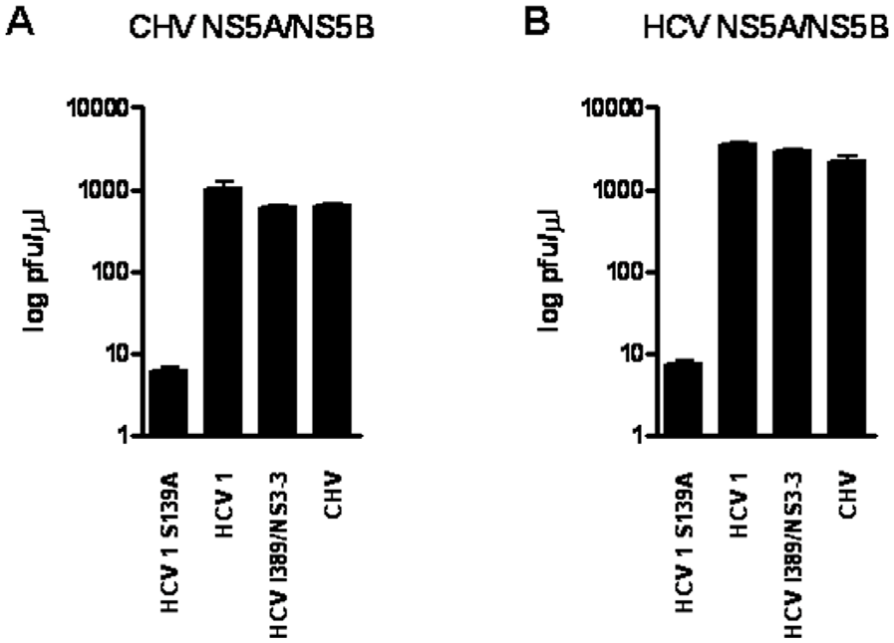
Comparative catalytic efficiencies of HCV and CHV NS3/4A proteases based on (A) CHV NS5A/5B and (B) HCV NS5A/5B viral polyprotein cleavage. The graph illustrates the resulting lambda phage titer (in PFU per microliter) after selective growth of lambda in *E. coli* cells coexpressing the NS3/4A protease constructs and the lambda cI repressor expressing either CHV NS5A/5B or HCV NS5A/5B viral polyprotein cleavage sites. Values are the means ± standard deviations (error bars) of at least three experiments.

**Figure 6:**
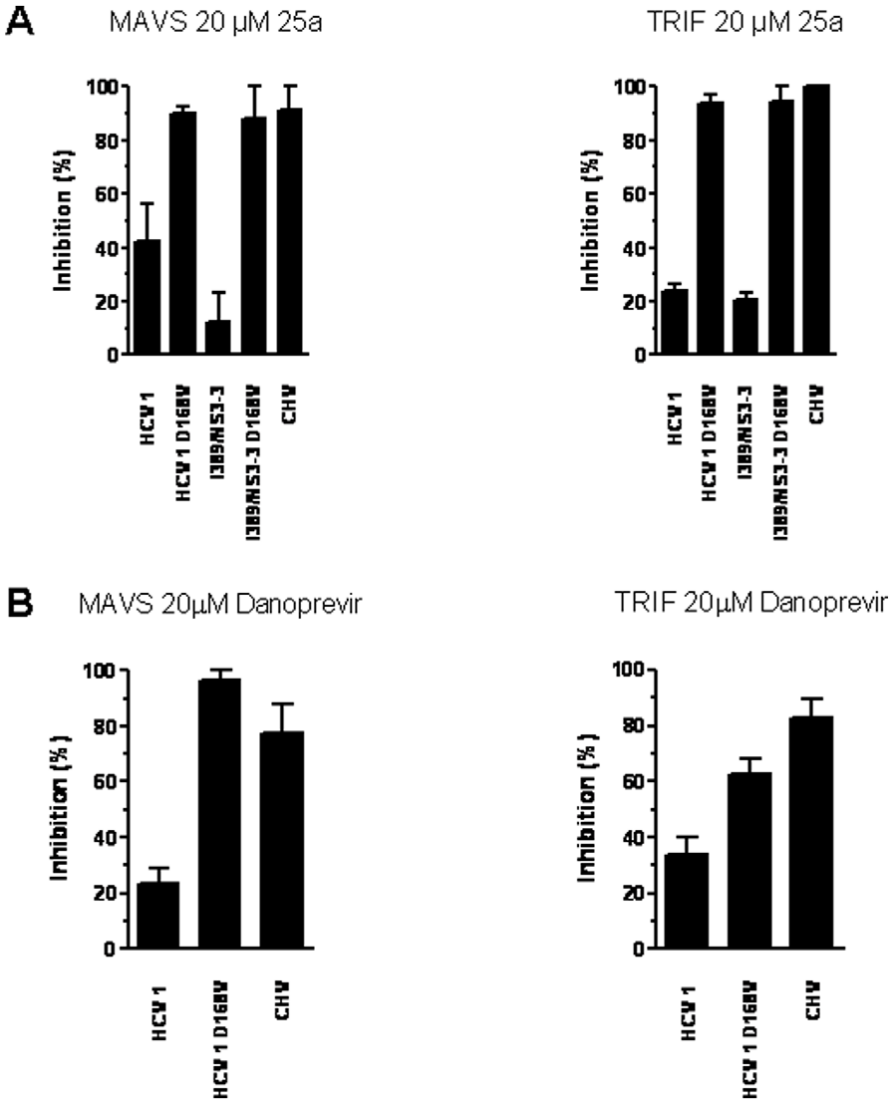
Inhibition of HCV and CHV NS3/4A proteases in the presence of the protease inhibitors (A) 25a or (B) danoprevir. The graph illustrates the relative (%) lambda phage titer after selective growth of lambda in *E. coli* cells coexpressing the NS3/4A protease constructs and the lambda cI repressor expressing either MAVS or TRIF cleavage sites in the absence or presence of protease inhibitors (20 µM). No significant inhibition was detected with an HCV NS3/4A protease mutant carrying the substitution D168V, which confers resistance to protease inhibitors. Values are the means ± standard deviations (error bars) of at least three experiments.

We next investigated whether CHV and HCV NS3/4A proteases could specifically process heterologous viral polyprotein cleavage sites. To do so, the CHV and HCV NS5A/NS5B cleavage sites were engineered (Fig. 1) and tested against both proteases. Interestingly, the CHV and HCV NS3/4A proteases processed the heterologous viral cleavage sites with comparable efficiency (Fig. 5), suggesting that CHV and HCV NS3/4A proteases may share the same evolutionary linage. Finally, two macrocyclic HCV NS3/4A protease inhibitors, 25a [28] and danoprevir [29], were assayed to test their ability to inhibit CHV NS3/4A protease. Macrocyclic inhibitors were chosen because they have a higher potency against different HCV genotypes compared to linear inhibitors [30]. The inhibitory capability of these two macrocyclic inhibitors was assessed with the MAVS and TRIF cleavage sites. As expected, 25a and danoprevir inhibited HCV NS3/4A protease (Fig. 6) when tested with either cleavage site. In contrast, no significant inhibition was observed when either of the two inhibitors was assayed with the CHV NS3/4A protease. Of note, the positions associated with resistance to macrocyclic HCV NS3/4A protease inhibitors, HCV residues Q80, R155, A156, and D168 [31], are mutated in the CHV NS3/4A protease sequence (Fig. 2). The specificity of this inhibition was tested by including an HCV NS3/4A protease mutant carrying the protease inhibitor resistance substitution D168V; this substitution reduced the inhibitory capacity of the two tested protease inhibitors (Fig. 6).

## Discussion

Recent studies have identified viruses, in domestic dogs and horses, which are genetically very closely related to HCV [3], [4]. These findings suggest that HCV could have been introduced into the human population through contact with dogs and/or horses. Nevertheless, adaptation of CHV and NPHV to human cells would likely require these viruses to evade the human cellular innate immune response, which responds to viral pathogens by RNA composition–dependent activation of RIG-I and subsequent signalling via interferon regulatory factor (IRF)-3 [6]. The response to HCV infection is regulated by hepatic immune defences triggered by cellular RIG-I [14], [32]. In human liver cells, HCV interferes with RIG-I–dependent signalling to IRF-3 by NS3/4A-dependent cleavage of the crucial adaptor protein MAVS [14]. Likewise, TLR-3–dependent signalling to IRF-3 is ablated by cleavage of TRIF by the HCV NS3/4A protease [15]. In this study, we tested the ability of CHV NS3/4A protease to specifically cleave the human adaptor proteins MAVS and TRIF.

We found that CHV NS3/4A protease could process, with comparable efficiency, the specific MAVS and TRIF cleavage sites known to be processed in vivo by HCV NS3/4A protease [14], [15]. These findings extend previous reports [3], [4], confirming a possible evolutionary relationship between CHV and HCV. The results of our study indicate that CHV can disrupt the human innate antiviral defense signaling pathway. In addition, our study also showed that, similar to HCV NS3/4A protease, CHV NS3 protease efficiency is regulated by the viral NS4 protein [33], suggesting that the mechanism of action and substrate specificity is similar between the two proteases. CHV NS4 polypeptide improved cleavage of MAVS- and TRIF-specific cleavage sites. However, CHV NS3/4A protease was not susceptible to macrocyclic inhibitors of HCV NS3/4A protease. Important HCV genotype–specific differences in the efficacy of NS3/4A protease inhibitors were reported recently [29]. Most of the HCV NS3/4A protease inhibitors now in clinical development were identified using HCV replicon systems of genotype 1 and are being licensed for treatment of genotype 1 infection. In vitro studies performed with HCV chimeras have shown that genotypes 2a, 5a, and 6a had similar sensitivity, whereas genotype 3a was comparatively resistant to macrocyclic HCV NS3/4A protease inhibitors [30]. CHV NS3/4A protease has amino acid changes at positions associated with resistance to macrocyclic HCV NS3/4A protease inhibitors [31], which would explain why this protease is resistant to macrocyclic HCV NS3/4A protease inhibitors.

GB virus B (GBV-B) is another hepacivirus that before the discovery of CHV/NHV was the virus phylogenetically most closely related to HCV. GBV-B does not infect humans or chimpanzees, but it causes acute hepatitis in experimentally infected tamarins (a type of New World monkey) [2]. Although there are important differences in the outcomes of HCV and GBV-B infections (GBV-B rarely causes persistent infections), GBV-B has evolved a strategy similar to that employed by HCV, resulting in the disruption of RIG-I signalling. Indeed, GBV-B NS3/4A proteolytically cleaves human MAVS. More distantly related hepacivirus such as GBV-C, which infects humans and chimpanzees, and GBV-A are also able to antagonizing MAVS [16]. In contrast, proteases from the more distantly related pestivirus bovine viral diarrhea virus and flavivirus yellow fever virus do not antagonize MAVS [16], [34]. Overall these data suggest that the ability of NS3/4A proteases to antagonize MAVS is a phylogenetically discrete characteristic exclusive to HCV and the three GBV viruses. Based on these observations, CHV NS3/4A should be able to cleave the canine's MAVS and TRIF proteins. Canine orthologs of human MAVS and TRIF differ in sequence at the cleavage site processed by HCV NS3/4A protease; therefore, they were not tested in this study. Inspection of the canine MAVS and TRIF proteins did not reveal the presence of apparent substrate specificities for HCV NS3/4A protease. Although CHV was initially found in dogs, subsequent efforts to find similar viruses in canids has remained largely unsuccessful [4]. Recently, evidence of a CHV-like virus infection was confirmed by the detection of diverse viral genomes, termed NPHV, in horses [4]. Horses can support replication of several other flaviviruses, including the vector-borne West Nile, Japanese encephalitis, Dengue, and St. Louis encephalitis viruses. These flaviviruses can be transmitted among several mammalian species, including humans. Most of the NPHV strains detected in horses are genetically distinct from CHV; however, one variant is almost identical to CHV, suggesting that CHV/NPHV viruses may be able to jump species [4].

This is the first study to our knowledge to demonstrate that at least parts of the identified and sequenced CHV/NPHV RNA genomes are biologically active. Initial attempts to culture CHV were not successful [3], [35]. Only a single strain of HCV, the genotype 2a strain JFH1 from a Japanese patient, has been found to grow robustly in culture, in human hepatoma cell lines [36]. However, viable JFH1-based recombinants with gene elements specific to other HCV genotypes have been developed [30], [37], [38]. Given the difficulties in culturing HCV, our results suggest that it may be possible to generate HCV/CHV chimeras that can be cultivated.

Our study has some limitations that are worth noting. First, this study only tested the functionality of a small CHV genomic region; other CHV/NPHV genomic regions that have been previously described [3], [4] should be analyzed to guarantee the viability of future HCV/CHV chimeras. Second, the in vitro approach used here to measure the ability of the protease to cleave human adaptors MAVS and TRIF only partially mimics what occurs in vivo. Third, it has not been determined here whether CHV NS3/4A protease can efficiently cleavage the MAVS and TRIF proteins equivalent of horses and canines, their primary hosts. Nevertheless, the similarity observed between HCV and CHV NS3/4A proteases in their capability to process MAVS and TRIF, as well as HCV/CHV NS5A/NS5B cleavage sites, strongly supports the hypothesis that CHV might be able to disrupt the human innate antiviral defense signaling pathway. Further work should include an evaluation of ability of the CHV NS3/4A protease to process both the human and canine adaptor proteins MAVS and TRIF in human and canine hepatoma cell lines, as well as the construction of HCV/CHV chimeras to explore this hypothesis or to perform other functional studies related to hepacivirus pathogenesis and treatment.

## Materials and Methods

Insertion of the MAVS and HCV NS5A/NS5B–specific cleavage sites (Fig. 1) into the lambda cI repressor has been described elsewhere [20], [24]. In this study, TRIF and CHV NS5A/NS5B–specific cleavage sites (Fig. 1) were inserted into the lambda cI repressor. Briefly, the nucleotide sequence for TRIF and CHV NS5A/NS5B–specific cleavage sites (Fig. 1) was chemically synthesized (Integrated DNA Technologies). The constructs included two restriction sites, NsiI and HindIII, at the ends, which are present in the lambda cI repressor [39]. After digestion with NsiI and HindIII, the construct was ligated into pcI.HCVNS5A/NS5Bcro [20], previously digested with NsiI and HindIII, to generate the pcI.TRIFcro and pcI.CHVNS5A/NS5Bcro plasmids Sequence analysis of this plasmid confirmed the accuracy of the synthetic construct. *E. coli* JM109 cells containing plasmid pcI.MAVScro, pcI.TRIFcro, pcI.HCVNS5A/NS5Bcro or pcI.CHVNS5A/NS5Bcro were then transformed with the plasmid expressing the corresponding protease. Transformed cells were grown overnight at 30°C in the presence of 0.2% maltose, 12.5 µg/ml tetracycline, and 20 µg/ml ampicillin; harvested by centrifugation; and resuspended to an optical density at 600 nm of 2.0/ml in 10 mM MgSO4. To induce expression of the protease, 20 µl of cells were incubated in 100 µl of Luria-Bertani (LB) medium containing 12.5 µg/ml tetracycline, 20 µg/ml ampicillin, 0.2% maltose, 10 mM MgSO4, and 0.1 mM isopropyl-β-D-thiogalactopyranoside (IPTG) for 1 h. The cell cultures were then infected with 10^5^ plaque-forming units (PFU) of phage. After 3 h at 37°C, the titer of the resulting phage was determined by coplating the cultures with 200 µl of *E. coli* XL-1 Blue cells (adjusted to an optical density at 600 nm of 2.0/ml in 10 mM MgSO4) on LB plates using 3 ml of top agar containing 12.5 µg of tetracycline/ml, 0.2% maltose, and 0.1 mM IPTG. After incubation at 37°C for 6 h, the resulting phage plaques were counted in order to score growth.

Western blots were performed as previously described [19]. Briefly, *E. coli* JM109 cells containing plasmid pcI.MAVScro or pcI.TRIFcro were transformed with the plasmid expressing the corresponding protease. Transformed cells were then grown overnight at 30°C in the presence of 0.2% maltose, 12.5 µg/ml tetracycline, and 20 µg/ml ampicillin; harvested by centrifugation; and resuspended to an optical density at 600 nm of 2.0/ml in 10 mM MgSO4. To induce expression of the protease, 200 µl of cells were incubated in 1 ml of LB medium containing 12.5 µg/ml tetracycline, 20 µg/ml ampicillin, 0.2% maltose, 10 mM MgSO4, and 0.1 mM IPTG for 3 h. The ODs of the cultures after 3 h (in the presence of IPTG) were measured to assure that equivalent amounts of total cell protein were blotted. No significant differences were observed when the ODs of the different cultures were compared, suggesting that the expression of the NS3/4A proteases did not affect the growth of the bacteria. Cultures were lysed in sodium dodecyl sulfate (SDS)-polyacrylamide gel electrophoresis sample buffer, resolved in 12% gradient SDS polyacrylamide gels (Invitrogen), transferred to nitrocellulose membranes, and blocked in phosphate-buffered saline–0.1% Tween 20–10% nonfat dry milk. For immunochemical detection of the lambda repressor, membranes were subsequently incubated with rabbit serum containing polyclonal anti-cI antibodies (anti-cI sera; Invitrogen). Bound antibodies were visualized with peroxidase-linked anti-rabbit antybody HRP-linked IgG (Cell Signaling Technology) and the SuperSignal West Pico Chemiluminescent Substrate (Thermo Scientific).

The nucleotide sequence for CHV NS3/4A protease (Fig. 2) was chemically synthesized (Integrated DNA Technologies) following the nucleotide sequence reported by Kapoor et al. [3]. The construct included two restriction sites, EcoRI and XhoI, at the ends. After digestion with EcoRI and XhoI, the construct was cloned into pBluescript SK– (Agilent Technologies) to generate a β-galactosidase–CHV NS3/4A protease fusion protein. Sequence analysis of this plasmid confirmed the accuracy of the synthetic construct. Control mutant constructs (red residues in Figs. 1 and 2) were generated by site-directed mutagenesis using the QuikChange kit (Agilent Technologies) and following the manufacturer's instructions. Three HCV NS3/4A proteases used in this study, 1, 50, and 51, were obtained from HCV genomic RNA extracted from the plasma of three different HCV-infected patients. The nucleotide sequences and amplification procedures of these proteases have been published previously [18], [24]. The fourth HCV NS3/4A protease employed in this study was amplified from the HCV subgenomic replicon I389/NS3-3 [26]. Macrocyclic protease inhibitor 25a [28] was kindly provided by Merck, Sharp and Dohme. Donoprevir was purchased from Selleck Chemicals (Houston, TX, USA).
